# A Rapid Increase in Serum Lactate Levels after Cardiovascular Surgery Is Associated with Postoperative Serious Adverse Events: A Single Center Retrospective Study

**DOI:** 10.3390/diagnostics14182082

**Published:** 2024-09-20

**Authors:** Kenichiro Kikuchi, Satoshi Kazuma, Yoshiki Masuda

**Affiliations:** 1 Department of Anesthesiology, Japanese Red Cross Kitami Hospital, Kitami 090-8666, Japan; kikuchi1@sapmed.ac.jp; 2 Department of Intensive Care Medicine, School of Medicine, Sapporo Medical University, Sapporo 080-8543, Japan; miminekko@me.com

**Keywords:** lactate, hyperlactatemia, cardiovascular surgery, postoperative serious adverse event (PSAE)

## Abstract

Background/Objectives: Hyperlactatemia is a common predictive factor for poor post-cardiovascular surgery outcomes. However, it is not well understood whether the rapid postoperative lactate level elevation in a short period of time is associated with patient outcomes. Herein, we investigated the relationship between the degree of change in serum lactate levels and postoperative serious adverse events (PSAEs), including mortality, within 24 h of cardiovascular surgery. Methods: In this retrospective study, we evaluated the relationship between a rapid serum lactate level increase and PSAEs after open-heart and major vascular surgery. We divided the patients into those with and without PSAEs. Univariate and multivariate analyses were performed to evaluate the association between PSAEs and rapid lactate level increases. Results: We enrolled 445 patients; 16% (n = 71) had PSAEs. The peak lactate levels during the first 24 h of intensive care unit (ICU) stay were higher in patients with PSAEs than in those without. The maximum change in lactate levels between two consecutive lactate measurements during the first 24 h after ICU admission was higher in patients with PSAEs than in those without. A multivariate logistic regression analysis revealed that changes in lactate levels of 2 mmol/L or more between two consecutive lactate measurements were associated with PSAEs. ICU peak lactate levels of 3 mmol/L or more were not associated with PSAEs. Conclusions: Rapid serum lactate level increases of 2 mmol/L or more during the first 24 h of ICU admission post-cardiovascular surgery are associated with PSAEs.

## 1. Introduction

Hyperlactatemia is widely recognized as a significant indicator of poor outcomes during and after cardiovascular surgery [1,2,3]; regardless of the cause, patients with hyperlactatemia have a poor prognosis [4]. However, there is no consensus on the threshold of high lactate levels for predicting outcomes in patients undergoing cardiovascular surgery. In addition, the absolute value of hyperlactatemia is a static indicator, making the early detection of rapid changes in the patient’s condition postoperatively difficult. Dynamic lactate indices were reported to predict the prognosis of critically ill patients better than static indices [5,6]. Thus, the dynamic change in lactate levels, rather than the absolute value of serum lactate and the timing of hyperlactatemia, may have an impact on serious complications after cardiovascular surgery. We hypothesized that a rapid change in serum lactate levels may better predict postoperative serious adverse events (PSAEs) than absolute high lactate levels within 24 h after cardiovascular surgery. In this study, we aimed to investigate the relationship between the degree of change in serum lactate levels and PSAEs, including mortality, within 24 h of cardiovascular surgery.

## 2. Materials and Methods

### 2.1. Patients

In this retrospective, observational, single-center study, we included patients admitted to the intensive care unit (ICU) of a single tertiary university medical center between January 2015 and December 2018 after cardiovascular surgery. We excluded patients with missing data, those who were not admitted to the ICU after surgery, those who underwent endovascular stent grafting surgery, or those who underwent abdominal aortic aneurysm surgery. Patients admitted after stent grafting and abdominal aortic surgery were excluded because they were not routinely admitted to the ICU. The included patients were divided into those with PSAEs and those without (no PSAEs). PSAEs were defined as the presence of one or more of the following: in-hospital death, need for reoperation, need for circulatory assistance devices, need for reintubation, need for dialysis, or ICU readmission. This study was approved by the Institutional Review Board of Sapporo Medical University (approval number: 312-148). Informed consent was not required owing to the retrospective nature of this study.

### 2.2. Data Collection

Patient information was obtained from hospital electronic medical records and ICU medical records. Anesthetic management during surgery was obtained from electronic anesthesia records. Blood lactate samples were collected intraoperatively in the operating theater and postoperatively in the ICU. During surgery, blood gas analyses, including lactate levels, were performed by drawing blood samples from an arterial catheter at the discretion of an anesthesiologist or clinical engineer. Blood gas analysis was conducted immediately after ICU admission and every 4 h thereafter, or at the discretion of the physician, until the patient was discharged from the ICU. Blood samples were collected in heparinized blood gas syringes and measured at 37 °C. All measurements were performed using a blood gas analyzer (ABL800 FLEX; Radiometer, Copenhagen, Denmark).

We collected the following preoperative variables: age, sex, comorbidities (hypertension, diabetes mellitus, or dyslipidemia), estimated glomerular filtration rate (eGFR), hemoglobin (Hb) level, priority of surgery (elective or urgent), and type of surgery. Preoperative renal impairment was defined as an eGFR < 60 mL/min/1.73 m^2^. Intraoperative variables included the duration of cardiopulmonary bypass (CPB), duration of cross-cramp, circulatory arrest time, transfusion volume, intraoperative blood salvage volume, administered albumin volume, bleeding volume, urine output, and peak lactate levels during anesthesia. Postoperative variables included lactate level at ICU admission, peak lactate level during the first 24 h after ICU admission, maximum amount of change in lactate level between two consecutive blood gas analyses during the first 24 h after ICU admission (ΔLac), length of mechanical ventilation, length of ICU stay, length of hospital stay, 28 d mortality, 90 d mortality, and hospital mortality. We investigated the cutoff value of the absolute peak lactate level and ΔLac during the first 24 h after ICU admission. The frequency of PSAEs was examined in groups with peak lactate levels <2, 2–<3, and ≥3 mmol/L during the first 24 h after ICU admission. The frequency of PSAEs was also examined in groups with ΔLac <1, 1–<2, and ≥2 mmol/L.

### 2.3. Outcome Measures

The primary outcome was the development of PSAEs, and we investigated whether PSAEs were associated with a rapid increase in serum lactate levels. A rapid increase in serum lactate levels was defined as ΔLac ≧ 2 mmol/L. We also investigated whether a peak lactate level ≥3 mmol/L during the first 24 h after ICU admission was a predictive factor for PSAEs.

### 2.4. Statistical Analysis

Categorical variables are presented as percentages and numbers and continuous variables as medians with interquartile ranges (IQRs). The chi-squared or Fisher’s exact test (for categorical variables) and Student’s *t*-tests or Mann–Whitney U test (for continuous variables) were used to test for between-group differences, as appropriate. We compared pre-, intra-, and postoperative variables between patients with and without PSAEs. We performed univariate and multivariate analyses using a logistic regression model to investigate the value of ΔLac ≧ 2 in predicting PSAEs. Univariate parameters (*p* < 0.05) or clinically important factors were used as confounding variables in the multivariate logistic regression analyses. Odds ratios were presented with 95% confidence intervals (CI), and statistical significance was set at *p* < 0.05. Statistical analyses were performed using the GraphPad Prism 10 software (Insight Partners; New York, NY, USA).

## 3. Results

### 3.1. Patient Characteristics

**Figure 1:**
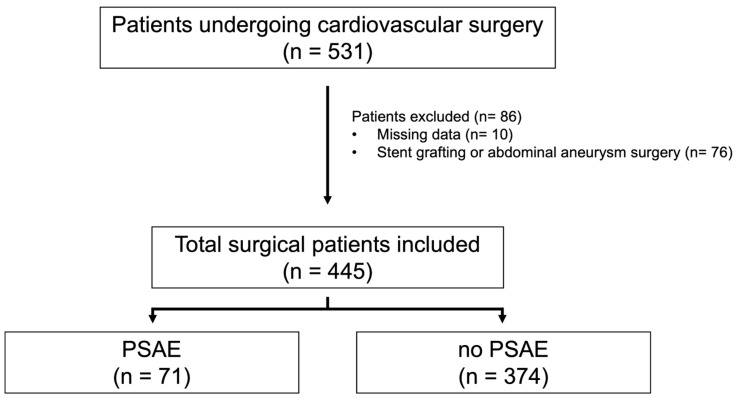
Flow diagram of study patients’ enrollment. Among 531 patients undergoing cardiovascular surgery, 445 were included for analysis in this study. Among them, 71 had PSAEs, and 374 did not. One or more of the following were considered PSAEs: in-hospital death, need for revision, need for circulatory assist devices, need for reintubation, need for dialysis, and intensive care unit re-entry. Abbreviation: PSAEs, postoperative serious adverse events.

In total, 531 patients who underwent cardiovascular surgery between January 2015 and December 2018 were assessed for eligibility. Of these, 445 (83.8%) were included in the analysis (Figure 1).

Regarding preoperative characteristics, patients with PSAEs had lower Hb levels than those without (median (IQR), 12.0 (10.7, 13.2) vs. 12.7 (11.5, 13.9) g/dL; *p* = 0.02). Patients with PSAEs had a higher frequency of urgent surgery (39.4% vs. 13.9%; *p* < 0.001) and major vascular surgery (66.2% vs. 48.9%; *p* = 0.0076) than those without. The CPB duration was longer in those with PSAEs than in those without (208 (146, 271) vs. 172 (116, 220) min; *p* < 0.001). Additionally, the transfusion and bleeding volumes were greater in patients with PSAEs. The peak lactate levels during the operation were higher in patients with PSAEs than in those without (3.1 (1.8, 5.0) vs. 2.1 (1.3, 3.3) mmol/L; *p* < 0.001). After ICU admission, there were significant differences in all variables evaluated in this study. The peak lactate levels were higher in patients with PSAEs than in those without (4.0 (2.9, 6.1) vs. 3.1 (2.3, 4.4) mmol/L; *p* < 0.001) (Table 1).

**Figure 2:**
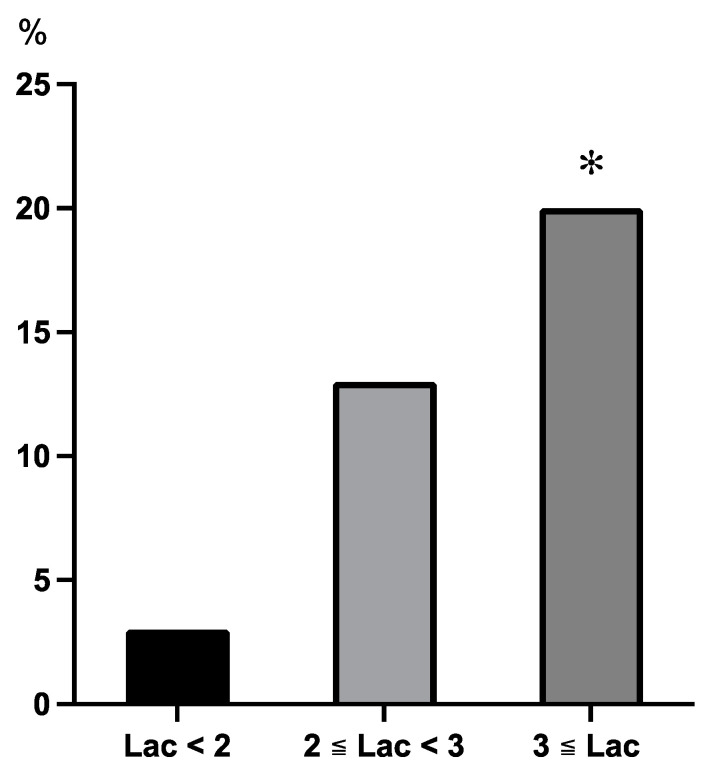
The incidence of PSAEs among subgroups based on the maximum Lac levels during the first 24 h after intensive care unit admission. The percentage of PSAEs in the Lac < 2 mmol/L, 2 ≦ Lac < 3 mmol/L, and Lac ≥ 3 mmol/L groups were 3%, 13%, and 20%, respectively. The percentage of PSAEs in the Lac ≥ 3 mmol/L group was significantly higher than that in the Lac < 2 mmol/L group. *: *p* < 0.001 vs Lac < 2 mmol/L group. Abbreviations: PSAE, postoperative serious adverse event; Lac, lactate.

The percentages of patients with PSAEs with maximum ICU lactate levels of <2, 2–3, and >3 mmol/L during the first 24 h in the ICU were 3.1%, 13.1%, and 20.5%, respectively. The percentage of patients with PSAEs with a maximum ICU lactate level of ≥3 mmol/L was significantly higher than that of patients with a level of <2 mmol/L (*p* < 0.001) (Figure 2).

**Figure 3:**
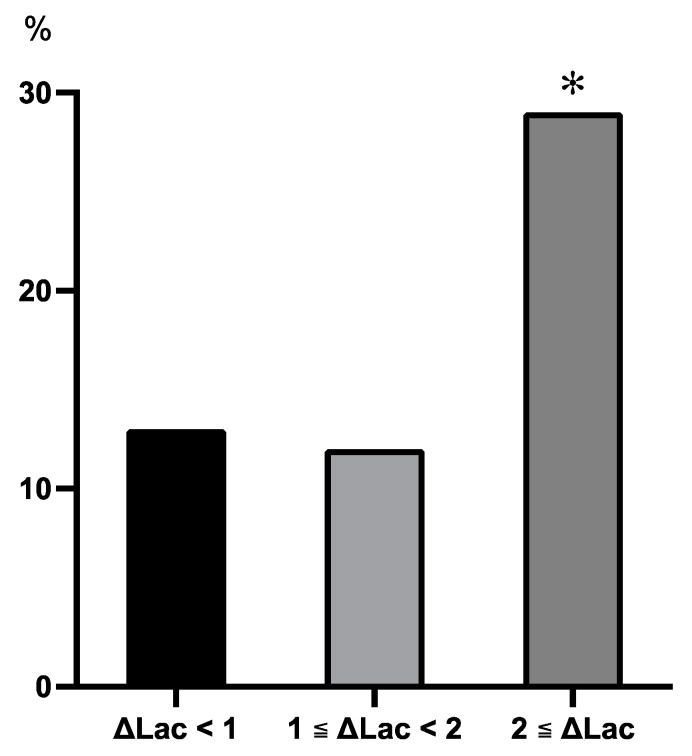
The incidence of PSAEs among subgroups based on the ΔLac in the first 24 h after intensive care unit admission. The percentages of PSAE in the ΔLac < 1 mmol/L, 1 mmol/L ≦ ΔLac < 2 mmol/L, and ΔLac ≥ 2 mmol/L groups were 13%, 12%, and 29%, respectively. The percentage of PSAE in the ΔLac ≥ 2 mmol/L group was higher than that in the ΔLac < 1 mmol/L and 1 mmol/L ≦ ΔLac < 2 mmol/L groups. *: *p* < 0.05 vs ΔLac < 1 mmol/L and 1 mmol/L ≦ ΔLac < 2 mmol/L groups. Abbreviations: PSAE, postoperative serious adverse event; Lac, lactate.

The incidence rates of PSAEs in the ΔLac < 1 mmol/L, 1 mmol/L ≦ ΔLac < 2 mmol/L, and ΔLac ≥ 2 mmol/L groups were 13.1%, 11.8%, and 28.9%, respectively. The incidence of PSAEs increased by more than twofold in the ΔLac ≦ 2 mmol/L group compared with the other groups. The incidence of PSAEs in the ΔLac ≦ 2 mmol/L group was greater than that in the ΔLac < 1 mmol/L and 1 mmol/L ≦ ΔLac < 2 mmol/L groups (*p* = 0.016 and *p* = 0.011, respectively) (Figure 3).

### 3.2. Outcomes

**Figure 4:**
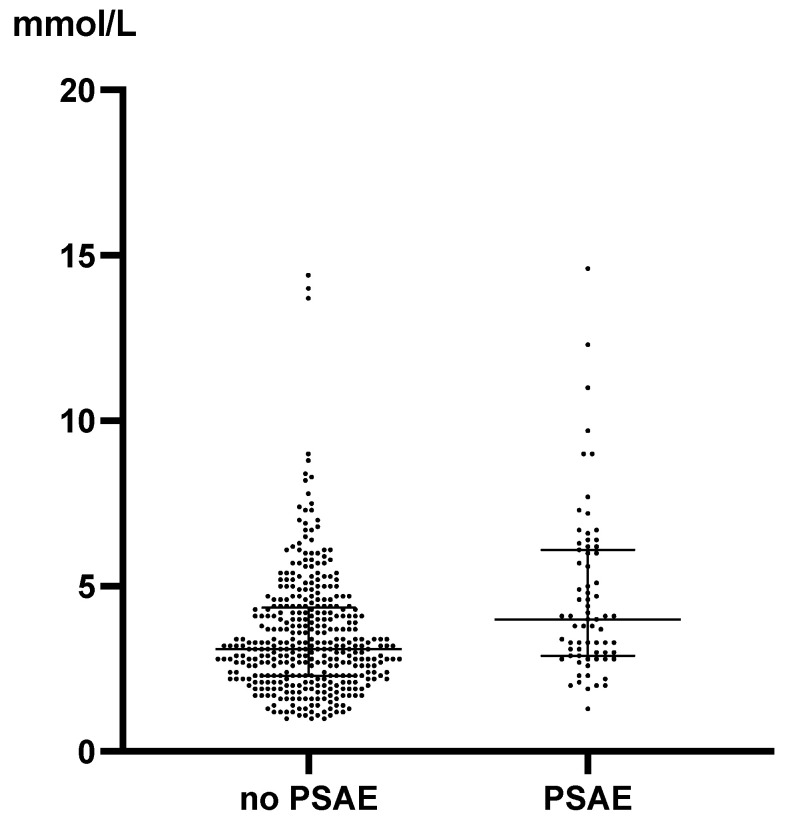
The peak Lac levels during the first 24 h after intensive care unit admission. The peak Lac levels during the first 24 h after ICU admission were lower in the group without PSAEs than in the group with PSAEs (3.1 (2.3–4.4) vs. 4.0 (2.9–6.1); *p* < 0.0001). The dots indicate individual patients, and the horizontal lines indicate the median with interquartile range. Abbreviations: PSAE, postoperative serious adverse event; Lac, lactate.

The peak lactate levels during the first 24 h of ICU stay were higher in the group with PSAEs than in the group without PSAEs (4.0 (2.9, 6.1) vs. 3.1 (2.3, 4.4); *p* < 0.001) (Figure 4).

**Figure 5:**
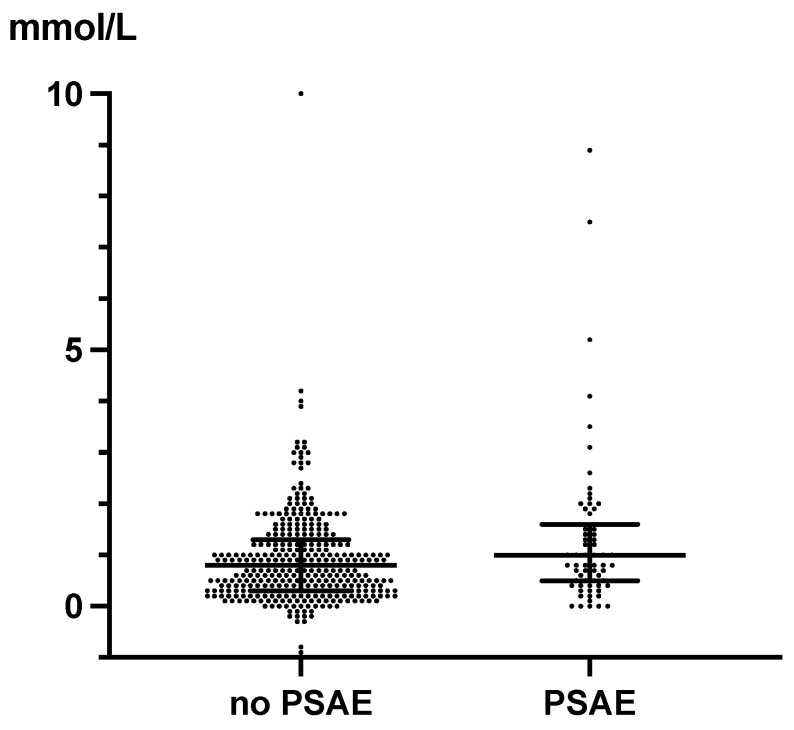
ΔLac during the first 24 h after intensive care unit admission. The ΔLac in patients with PSAEs was significantly higher than in patients without PSAEs (1.0 (0.5–1.6) vs. 0.8 (0.3–1.3); *p* = 0.02). The dots indicate individual patients, and the horizontal lines indicate the median with interquartile range. Abbreviations: PSAE, postoperative serious adverse event; Lac, lactate.

ΔLac was higher in the group with PSAEs than in the group without PSAEs (1.0 (0.5, 1.6) vs. 0.8 (0.3, 1.3); *p* = 0.02) (Figure 5).

In the univariate logistic regression analysis, urgent surgery, main vascular surgery, CPB duration, ICU peak Lac ≧ 3 mmol/L, and ΔLac ≧ 2 mmol/L were associated with PSAEs. In the multivariate logistic regression analysis, ΔLac ≧ 2 mmol/L was associated with PSAEs (odds ratio (OR), 2.35; 95% CI, 1.04–5.31; *p* = 0.04). CPB duration and urgent surgery were also associated with PSAEs (OR, 1.00; 95% CI, 1.00–1.01; *p* < 0.01; OR, 3.45; 95% CI, 1.84–6.47; *p* < 0.01, respectively). On the other hand, ICU peak-Lac ≧ 3 mmol/L in the ICU was not independently associated with PSAEs (OR, 1.43; 95% CI, 0.75–2.75, *p* = 0.28) (Table 2).

## 4. Discussion

In this study, we investigated the relationship between the degree of change in serum lactate levels and PSAEs within 24 h of cardiovascular surgery. Our study shows that in patients undergoing cardiovascular surgery, the peak lactate level and ΔLac were significantly higher in patients with PSAEs than in those without. This study also shows that the magnitude of the change in lactate levels, rather than the absolute value of lactate levels, was an independent predictor of PSAEs.

The thresholds for hyperlactatemia and ΔLac were defined as 3 mmol/L and 2 mmol/L, respectively. Hyperlactatemia is often defined as a lactate level of 2 mmol/L, and a lactate level of 3 mmol/L at ICU admission or persistent lactate levels of 3 mmol/L after ICU admission were reportedly predictive factors of a poor prognosis after cardiac surgery [7,8]. We found that the percentage of PSAEs increased in proportion to the increase in lactate levels in the absolute lactate level subgroup analysis (Figure 2). Lactate levels of 3 mmol/L or greater were associated with a higher percentage of PSAEs than lactate levels of 2 mmol/L. The percentages of PSAEs in the ΔLac < 1 mmol/L and 1 mmol/L ≦ ΔLac < 2 mmol/L subgroups were almost equal. The subgroup with ΔLac ≧ 2 mmol/L had a significantly higher percentage of PSAEs than the other groups (Figure 3). Notably, a change in lactate level greater than 2 mmol/L after extracorporeal circulation has been associated with a prolonged ICU stay and an increased incidence of PSAEs in pediatric patients after cardiac surgery [9]. A ΔLac threshold of 2 mmol/L was considered appropriate because ΔLac ≧ 2 mmol/L showed a large increase in the incidence of PSAEs in this study. 

Patients with PSAEs had higher peak lactate levels and greater short-term increases in lactate levels than those without within 24 h of ICU admission (Figure 4 and Figure 5). The absolute level of hyperlactatemia and persistent hyperlactatemia are reportedly associated with poor patient outcomes [8]. Hyperlactatemia is the result of increased lactate production, decreased lactate clearance, or a combination of both [5]. Hyperlactatemia in patients undergoing cardiovascular surgery can be caused by both hypoxic and non-hypoxic mechanisms [2]. The lactate/pyruvate ratio is useful in differentiating hypoxic from non-hypoxic hyperlactatemia. An increase in the lactate/pyruvate ratio indicates tissue hypoxia [2] and is associated with higher mortality than a normal lactate/pyruvate ratio [10]. However, distinguishing the mechanisms of hyperlactatemia is difficult due to tissue hypoxia because serum pyruvate cannot be measured using a conventional blood analysis. Patients with hypoxia often present with local or systemic hypoperfusion resulting from cardiopulmonary arrest, hypovolemic shock, sepsis, or heart failure. Systemic hypoperfusion was not observed in patients with non-hypoxic hyperlactatemia. Causes of non-hypoxic hyperlactatemia include liver disease, malignancy, human immunodeficiency virus infection, alcoholism, and drug use [11,12]. Adrenergic beta-receptor stimulation or CPB is a cause of non-hypoxic hyperlactatemia in the perioperative period of cardiovascular surgery. Hyperlactatemia in the immediate postoperative period is induced by surgical procedures such as CPB, circulatory arrest, or catecholamine administration during surgery. In this study, we did not evaluate the causes of hyperlactatemia and a rapid increase in lactate levels. However, hyperlactatemia and a rapid increase in lactate levels within 24 h of ICU admission suggested that patients were likely to develop PSAEs. These findings suggest the need for early investigation of the cause and intervention when these findings are observed.

The univariate logistic regression analysis results show that urgent surgery, major-vascular surgery, the duration of CPB, the peak lactate level in the ICU, and ΔLac ≧ 2 mmol/L are predictive factors of PSAEs. In the multivariate logistic regression analysis, urgent surgery, CPB, and ΔLac ≧ 2 mmol/L were found to be predictive factors for PSAEs. Thus, the magnitude of change in lactate levels was a predictor of PSAEs, whereas the absolute lactate levels were not an independent predictor of PSAEs in this study. The degree, duration, and timing of hyperlactatemia after cardiovascular surgery were reported to be poor prognostic factors for patients [3,8,13]. Changes in lactate levels after CPB were also reported as poor postoperative prognostic factors in pediatric cardiac surgery [9]. The greater the absolute value of hyperlactatemia or the change in lactate levels, the more likely the patients were to undergo PSAEs. A total of 75% of patients with and 55% of patients without PSAEs had a lactate level of 3 mmol/L or higher during the first 24 h after ICU admission (*p* = 0.0024). Although the proportion of patients with hyperlactatemia was significantly higher in patients with PSAEs, more than half of the patients in both groups had hyperlactatemia. Nevertheless, the timing of the peak lactate levels in each patient was not evaluated in this study. The time course of lactate dynamics after cardiovascular surgery varies. The results could have been different if the timing of the peak lactate levels within 24 h of admission to the ICU were different. A large change in lactate levels was associated with PSAEs at any time within 24 h of ICU admission. A rapid decrease in lactate levels suggests improved lactate clearance [14]. Conversely, a rapid increase in lactate level suggests increased lactate production or decreased lactate clearance. The rate of change in lactate levels after the return of spontaneous circulation is also associated with mortality and neurological outcomes in patients after cardiac arrest [15]. Changes in lactate levels, rather than absolute lactate levels, can provide real-time information regarding changes in a patient’s condition after cardiovascular surgery. The dynamic evaluation of lactate levels may be more useful than a single value in assessing the prognosis of critically ill patients. Decreased lactate levels may indicate the success of therapeutic interventions. In a previous study, among the subgroups of patients in the ICU, those with continuously decreasing lactate levels had a better prognosis [16]. However, an increase in lactate levels indicates an imbalance between lactate production and metabolism. These conditions indicate the failure of therapeutic intervention or onset of impaired lactate clearance, such as heart failure, acute hemorrhage, and non-occlusive mesenteric ischemia [3]. A rapid increase in lactate levels could indicate rapid deterioration of the patient’s condition and could be a factor leading to PSAEs. In this study, we show that patients with PSAEs had a higher degree of lactate elevation than those without. The rapid increase in serum lactate levels was useful for identifying patients at risk of PSAEs. These patients require careful observation and aggressive interventions.

This study had several limitations. First, as a retrospective single-center study, it may be difficult to extrapolate the findings to other patients in a similar setting. Second, the cause of hyperlactatemia was not evaluated in this study. Understanding the pathophysiology of elevated lactate levels may allow us to more accurately examine the relationship between elevated lactate levels and postoperative complications. Third, we did not collect information that may have affected the postoperative lactate levels. As this is a retrospective study, the initiation and dose adjustment of catecholamines and vasoactive agents were left to the “discretion” of the anesthesiologist, intensivist, and cardiac surgeon and were not adjusted according to a fixed protocol, which may complicate the interpretation of the results. In addition, the relationship between vasoconstrictors such as norepinephrine is not constant, and it is possible that it affects both microcirculation and macrocirculation, which may complicate the interpretation of results of this study. Therefore, the acquisition of data on catecholamine administration was not investigated in this study. Fourth, the definitions of hyperlactatemia and a rapid increase in lactate levels may be inappropriate. We set the threshold for hyperlactatemia at 3 mmol/L and that for a rapid increase in lactate level at 2 mmol/L. This value was relatively low for hyperlactatemia in previous studies. The threshold of this value has high sensitivity but may have low specificity for predicting PSAEs.

## 5. Conclusions

The peak lactate levels and rapid changes in lactate levels during the first 24 h after cardiovascular surgery were higher in patients with PSAEs than in those without. In the multivariate logistic regression analyses, hyperlactatemia of 3 mmol/L or more during the first 24 h after cardiovascular surgery was not independently associated with PSAEs. A rapid increase in serum lactate of 2 mmol/L or more was independently associated with PSAEs.

## Figures and Tables

**Table 1:** Baseline characteristics of study population.

Variables	PSAEs (n = 71)	No PSAEs (n = 374)	*p*
Preoperative			
Sex (% male)	60.6	60.4	>0.99
Age (y) (IQR)	70 (62–77)	71 (64–76)	0.94
Hypertension (%)	56.3	56.8	0.94
Diabetes mellitus (%)	18.3	23.3	0.35
Dyslipidemia (%)	16.9	23.8	0.20
eGFR (% <60 mL/min/1.73 m^2^)	53.5	46.8	0.30
Hb (g/dL) (IQR)	12 (10.9–13.2)	12.7 (11.5–13.9)	0.02
Urgent surgery (%)	39.4	13.9	<0.001
Type of surgery			
Single valve surgery (n) (%)	7 (9.0)	71 (91.0)	
Single valve surgery + CABG (n) (%)	6 (18.2)	27 (81.8)	
Multiple valve surgery (n) (%)	5 (15.6)	27 (84.4)	
Multiple valve surgery + CABG (n) (%)	3 (33.3)	6 (66.7)	
CABG (n) (%)	0 (0)	3 (100)	
Thoracic aortic surgery (n) (%)	45 (19.7)	183 (80.3)	
Other surgery (n) (%)	5 (8.2)	56 (91.8)	
Intraoperative			
CPB (min) (IQR)	208 (146–271)	172 (116–220)	<0.001
Cross-clamp (min) (IQR)	94 (40–156)	93 (0–140)	0.28
Circulatory arrest (min) (IQR)	0 (0–57)	0 (0–49.75)	0.098
RBC (mL) (IQR)	1960 (840–3360)	1120 (560–1820)	<0.001
FFP (mL) (IQR)	1440 (960–2160)	960 (480–1440)	<0.001
PC (mL) (IQR)	250 (250–250)	250 (0–250)	<0.001
Blood salvage (mL) (IQR)	0 (0–0)	0 (0–460)	0.0073
Albumin (mL) (IQR)	0 (0–500)	0 (0–500)	0.061
Hemorrhage (mL) (IQR)	2300 (830–3430)	1535 (565–2500)	0.0022
Urine (mL) (IQR)	1100 (475–1800)	1000 (626–1596)	0.72
Peak Lac (mmol/L) (IQR)	3.1 (1.8–5.0)	2.1 (1.3–3.3)	<0.001
Postoperative			
Lac in ICU admission (mmol/L) (IQR)	2.7 (1.8–4.3)	2 (1.2–3.2)	<0.001
ICU peak Lac (mmol/L) (IQR)	4 (2.9–6.1)	3.1 (2.3–4.4)	<0.001
ICU ΔLac (mmol/L) (IQR)	1.0 (0.5–1.6)	0.8 (0.3–1.3)	0.02
Mechanical ventilation (d) (IQR)	2 (2–4)	1 (1–2)	<0.001
ICU stay (d) (IQR)	3 (2–5)	2 (2–2)	<0.001
Hospital stay (d) (IQR)	35 (24–59)	22 (18–29)	<0.001
28 d mortality (%) (n)	7.3 (5)	0.3 (1)	<0.001
90 d mortality (%) (n)	12.7 (8)	0.9 (3)	<0.001

Abbreviations: PSAE, postoperative serious adverse event; IQR, interquartile range; eGFR, estimated glomerular filtration rate; Hb, hemoglobin; CABG, coronary artery bypass grafting; CPB, cardiopulmonary bypass; RBC, red blood cell; FFP, fresh frozen plasma; PC, platelet concentrates; Lac, lactate; ICU, intensive care unit.

**Table 2:** Logistic regression analysis for prediction of PSAEs.

	Univariate Analysis		Multivariate Analysis	
	Odds Ratio (95% CI)	*p*	Odds Ratio (95% CI)	*p*
Sex, male	1.0 (0.60–1.69)	0.98	1.12 (0.64–1.97)	0.70
Age	0.99 (0.98–1.02)	0.94	1.00 (0.97–1.02)	0.83
Urgent surgery	4.03 (2.31–8.05)	<0.01	3.45 (1.84–6.47)	<0.01
Type of surgery, vascular	2.04 (1.20–3.48)	<0.01	1.05 (0.56–1.96)	0.88
CPB	1.01 (1.00–1.01)	0.01	1.00 (1.00–1.01)	<0.01
ICU peak Lac ≧ 3 mmol/L	2.40 (1.36–4.26)	<0.01	1.43 (0.75–2.75)	0.28
ΔLac ≧ 2 mmol/L	2.61 (1.25–5.45)	0.01	2.35 (1.04–5.31)	0.04

Abbreviation: CI, confidence interval.

## Data Availability

The datasets generated and/or analyzed during this study are not publicly available because a research agreement from all authors is required for data sharing, but they are available from the corresponding author upon reasonable request.
